# Effect of Chitosan on Rhizome Rot Disease of Turmeric Caused by *Pythium aphanidermatum*

**DOI:** 10.1155/2014/305349

**Published:** 2014-03-06

**Authors:** Sathiyanarayanan Anusuya, Muthukrishnan Sathiyabama

**Affiliations:** Department of Plant Science, Bharathidasan University, Tiruchirappalli, Tamilnadu 620 024, India

## Abstract

Chitosan was evaluated for its potential to induce antifungal hydrolases in susceptible turmeric plant (*Curcuma longa* L.). Under field conditions, the application of chitosan (crab shell) to turmeric plants by foliar spray method induces defense enzymes such as chitinases and chitosanases. Such an increase in enzyme activity was enhanced by spraying chitosan (0.1% w/v) on leaves of turmeric plants at regular intervals. Gel electrophoresis revealed new chitinase and chitosanase isoforms in leaves of turmeric plants treated with chitosan. Treated turmeric plants showed increased resistance towards rhizome rot disease caused by *Pythium aphanidermatum*, whereas control plants expressed severe rhizome rot disease. Increased activity of defense enzymes in leaves of chitosan treated turmeric plants may play a role in restricting the development of disease symptoms. The eliciting properties of chitosan make chitosan a potential antifungal agent for the control of rhizome rot disease of turmeric.

## 1. Introduction

The rhizome rot disease caused by* Pythium aphanidermatum* is the most destructive disease of turmeric plants in India, which reduces its economic and commercial value [1, 2]. At present, effective fungicides are not available. Therefore, it is necessary to search for effective methods to control this pathogen. The possibility of stimulating internal plant defenses has become an interesting option for enhancing natural disease resistance. Higher plants have the ability to initiate various defense mechanisms when they are infected either by phytopathogens or after treatment with biotic and abiotic elicitors. Among the elicitors known to date, chitosan, a polyvalent cation, has been shown to act as a potent oligosaccharide elicitor which can induce defense responses in plant tissue [3]. Plant defense-related enzymes were known to participate in early defense mechanisms and to prevent pathogen infections [4, 5]. Chitosan has attracted tremendous attention as a potentially important biological resource due to its biological properties including biocompatibility, nontoxicity, and biodegradability [6]. Chitosan has been found to interfere with the growth of several plant pathogenic fungi [4, 7–9]. The potential of chitosan to protect fungal diseases of various horticultural plants has been studied in various investigations [4, 9–11]. The interest in the antimicrobial properties of chitosan was focused on its possible role in plant protection. Hence, the present study was carried out to induce resistance in susceptible turmeric plant (Erode local) against rhizome rot disease by application of chitosan.

## 2. Materials and Methods

### 2.1. Biological Material

Rhizomes of* Curcuma longa* (L.) cultivar Erode local (susceptible) were obtained from a farmer's field at Erode, Tamilnadu, India. The fungus* P. aphanidermatum* was obtained from ITCC (Indian Type Culture Collection), New Delhi, India, and was maintained on potato dextrose agar.

### 2.2. Preparation of Chitosan

1 g chitosan (crab shell, Sigma Chem. Co., USA) was dissolved in 40 mL of distilled water containing 9 mL of 1 M acetic acid. The pH was adjusted to 6.0 using sodium acetate. From this stock, 0.1% (w/v) chitosan solution was prepared and used for elicitor treatment.

### 2.3. Foliar Application of Chitosan

Rhizomes were thoroughly washed with running tap water thrice followed by glass distilled water; surface was sterilized by immersion in a sodium hypochlorite 0.001% (v/v) solution for 15 min followed by several rinses of sterile distilled water. For the field experiment, rhizomes were sown in plots and each plot had 30 plants, respectively, at a farmer's field, Erode district of Tamilnadu, India. For foliar spray, 30-day-old plants were treated with 0.1% chitosan (10 mL/plant) and the foliar spray was performed at a regular interval of 30 days up to 210 days. Water sprayed plants served as control. Each experiment was repeated three times.

### 2.4. Protein Extraction and Estimation

Leaf samples were collected at regular intervals and used for extraction. Turmeric leaves (1 g/2 mL) were homogenized with potassium phosphate buffer (0.02 M, pH 7.6) and centrifuged. The clear supernatant was used as a source of protein/defense enzyme. Protein content was determined by the method of [12] using BSA as standard.

### 2.5. Enzyme Assays

Chitinase was assayed by the method of Reissig et al. [13] using colloidal chitin as substrate. N-Acetyl glucosamine was used as standard. One unit of chitinase was defined as the amount of enzyme that liberated 1 *μ*mol of N-acetyl glucosamine per minute under assay condition.

Chitosanase activity was determined by the method of measuring the reducing sugars released from chitosan. The reducing sugars were estimated by the Nelson and Somogyi [14, 15] method using chitosamine HCl as standard. One unit of chitosanase was defined as the amount of enzyme that liberated 1 *μ*mol of reducing sugar as chitosamine per minute under assay condition.

### 2.6. Gel Electrophoresis

SDS-PAGE was carried out according to Laemmli [16]. Samples (50 *μ*g protein) were separated on 10% SDS-PAGE. For chitinase localization, 0.1% (w/v) glycol chitin was included in the separation gel. After electrophoresis, the gel was stained with calcofluor white M2R according to the procedure of Trudel and Asselin [17].

Chitosanase localization was carried out according to the method of Grenier and Asselin [18] in which the separation gel contains 0.02% glycol chitosan. After electrophoresis, the gel was stained with calcofluor white M2R.

### 2.7. Disease Severity

Control and chitosan treated plants (60-day-old) were challenged with 5 mL spores of* P. aphanidermatum* (1 × 10^5^ spores/mL). Disease severity was observed at different age levels.

### 2.8. Statistical Analysis

All the data were subjected to one-way analysis of variance to determine the significance of individual differences in *P* < 0.01 and 0.05 levels. All statistical analysis was conducted using SPSS 16 software support.

## 3. Results

### 3.1. Effect of Chitosan on Protein Content in Leaves of Turmeric Plant

**Figure 1:**
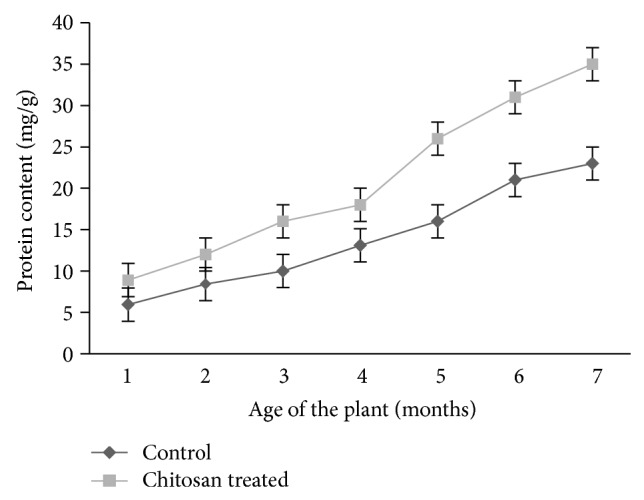
Protein content in control and chitosan treated leaves turmeric plants.

An increase in protein content in leaves of chitosan (0.1% w/v) treated plants was evident compared to control (water treated) plants (Figure 1).

### 3.2. Effect of Chitosan on Chitinase Activity

**Figure 2:**
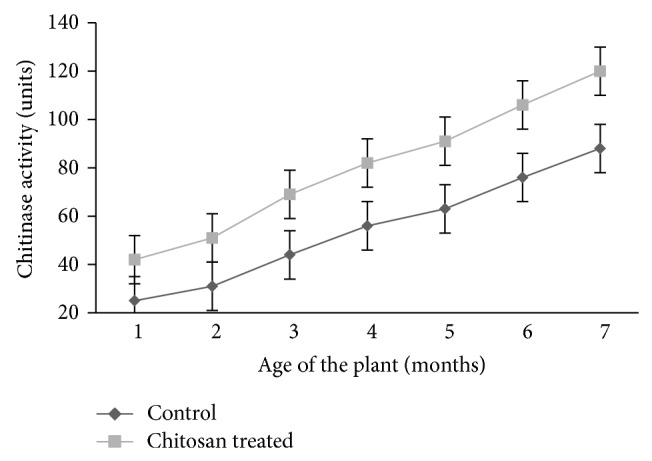
Chitinase activity in control and chitosan treated turmeric plants.

Turmeric plants treated with chitosan showed higher chitinase activity than untreated plants (Figure 2). There was a significant increase in chitinase activity in leaves of chitosan treated plants after 2 months and maximum chitinase activity was observed on the 7th month.

**Figure 3:**
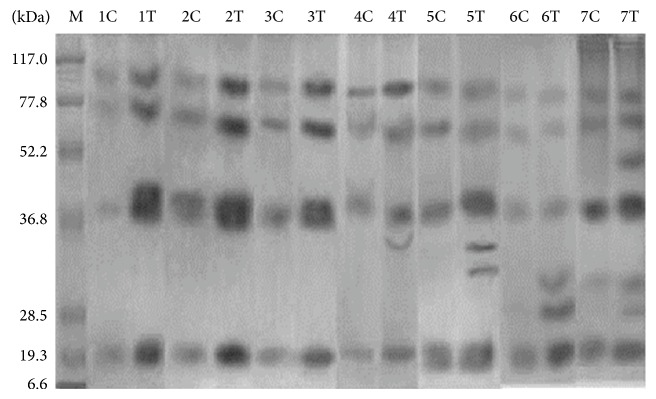
Localization of chitinase on SDS-PAGE. M—marker; C—control; T—treated; 1,2, 3,4, 5,6, and 7—age of the plant in months. Molecular weight of marker protein: 117.0 kDa—*β*-galactosidase; 77.8 kDa—BSA; 52.2 kDa—ovalbumin; 36.8 kDa—carbonic anhydrase; 28.5 kDa—soybean trypsin inhibitor; 19.3 kDa—lysozyme; 6.6 kDa—aprotinin.

On SDS-PAGE, different new isoforms of chitinase were observed in treated plants (Figure 3). In control plants, on the 1st month there were four constitutive chitinase polypeptides observed with molecular mass 110, 75, 37, and 19.3 kDa. These chitinase isoforms remained up to the 7th month. In chitosan treated plants, in the 1st month the induced constitutive chitinase of molecular mass 110, 75, 37, and 19.3 kDa was observed and these isoforms remained up to the 7th month. In addition to this, a new chitinase isoform of 35 kDa was observed in the 4th month after treatment. Apart from this, another new isoform of molecular mass 32 kDa was observed in 5th month, respectively. A new constitutive chitinase isoform of 26 kDa was observed in the 6th and 7th month, respectively (Figure 3).

### 3.3. Effect of Chitosan on Chitosanase Activity

**Figure 4:**
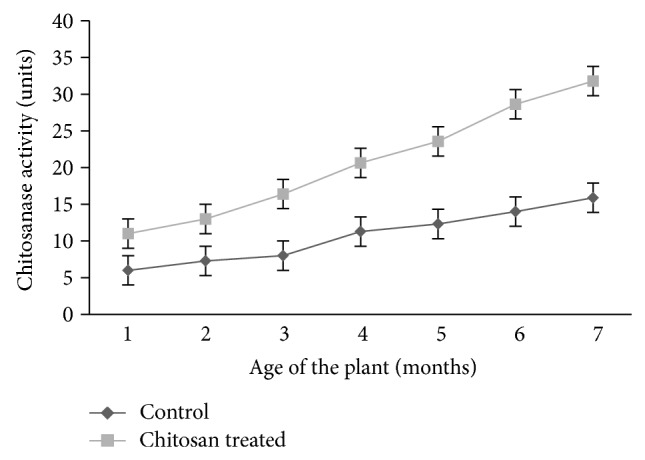
Chitosanase activity in control and chitosan treated turmeric plants.

Turmeric plants treated with chitosan showed an increase in chitosanase activity over a period of time and reached maximum on the 7th month. Nearly a threefold increase in chitosanase was observed in treated plants (Figure 4).

**Figure 5:**
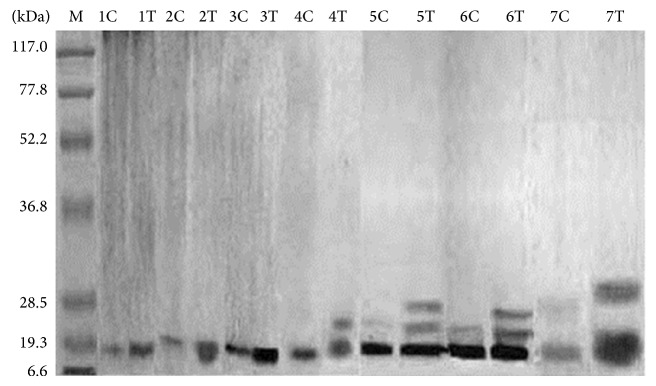
Localization of chitosanase on SDS-PAGE. M—marker; C—control; T—treated; 1,2, 3,4, 5,6, and 7—age of the plant in months. Molecular weight of marker protein: 117.0 kDa—*β*-galactosidase; 77.8 kDa—BSA; 52.2 kDa—ovalbumin; 36.8 kDa—carbonic anhydrase; 28.5 kDa—soybean trypsin inhibitor; 19.3 kDa—lysozyme; 6.6 kDa—aprotinin.

Chitosanase separated on SDS-PAGE showed new polypeptides in chitosan treated turmeric plants. In control plants, chitosanase polypeptide with molecular mass 19 kDa was observed in all the months. Apart from this, 23 kDa chitosanase polypeptide was observed in the 5th month of control plants. In chitosan treated plants, apart from the constitutive chitosanase polypeptide (19 kDa), a new isoform was observed after the 4th, 5th, 6th, and 7th month with molecular mass ranging from 23–30 kDa (Figure 5).

### 3.4. Effect of Chitosan on Disease Severity of Rhizome Rot Disease of Turmeric

**Figure 6:**
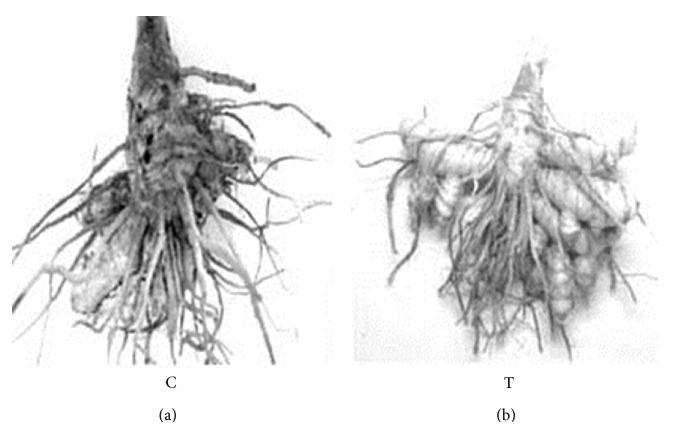
Turmeric rhizomes showing disease severity of rotting (C—control; T—treated).

Chitosan treated plants challenged with* P. aphanidermatum* showed a significant decrease in disease severity when compared to control (Figure 6).

## 4. Discussion

Inducing the plants own defense mechanisms by application of a biological inducer is thought to be a novel plant protection strategy. Chitosan can induce defense reactions in plants, including the induction of chitinase, chitosanase, and *β*-1,3 glucanase isoforms [19]. Chitosan and its derivatives offer a great potential as natural biodegradable substances which have antimicrobial and eliciting activities [5, 20]. Chitinase and chitosanase have been identified as PR proteins which might be implicated in plant defense system against pathogenic fungi [21, 22]. In the present study, we demonstrated that chitosan induced chitinase activity in leaves of turmeric plants. New chitinase isoforms were observed in treated plants. Chitosan stimulated chitinase production in cucumber plant and offered protection from root rot disease caused by* Pythium aphanidermatum *[18]. Celery,* Apium graveolens*, treated with chitosan showed a 20-fold increase in chitinase activity compared to that of chitosan-untreated plants and exhibited a delay in symptom expression caused by* F. oxysporum *[23]. It has been reported that a constitutive high level expression of chitinases in transgenic plants can enhance resistance to a variety of pathogens [24]. Prapagdee et al. [25] reported increased chitinase activity and the role of chitosan in protection of soybean. Our results could imply that the application of chitosan might sensitize the turmeric plant in protecting themselves from the phytopathogenic fungal invasion by elaboration of chitinase and chitosanase activity.

## 5. Conclusion

Chitosan played an important role in the growth suppression of* P. aphanidermatum* infection in turmeric plants. Increase in chitinase and chitosanase activity may play a role in enhanced resistance in turmeric plants against* P. aphanidermatum *infection.
